# Imaging Flow Cytometry as a Molecular Biology Tool: From Cell Morphology to Molecular Mechanisms

**DOI:** 10.3390/ijms26199261

**Published:** 2025-09-23

**Authors:** Yoshikazu Matsuoka

**Affiliations:** Department of iPS Stem Cell Regenerative Medicine, Kansai Medical University, Hirakata 573-1010, Osaka, Japan; matsuoka.yos@kmu.ac.jp; Tel.: +81-72-804-2393

**Keywords:** imaging flow cytometry, protein localization, signal transduction, cell cycle, immunological synapse, machine learning

## Abstract

Insights into the state of individual cells within a living organism are essential for identifying diseases and abnormalities. The internal state of a cell is reflected in its morphological features and changes in the localization of intracellular molecules. Using this information, it is possible to infer the state of the cells with high precision. In recent years, technological advancements and improvements in instrument specifications have made large-scale analyses, such as single-cell analysis, more widely accessible. Among these technologies, imaging flow cytometry (IFC) is a high-throughput imaging platform that can simultaneously acquire information from flow cytometry (FCM) and cellular images. While conventional FCM can only obtain fluorescence intensity information corresponding to each detector, IFC can acquire multidimensional information, including cellular morphology and the spatial arrangement of proteins, nucleic acids, and organelles for each imaging channel. This enables the discrimination of cell types and states based on the localization of proteins and organelles, which is difficult to assess accurately using conventional FCM. Because IFC can acquire a large number of single-cell morphological images in a short time, it is well suited for automated classification using machine learning. Furthermore, commercial instruments that combine integrated imaging and cell sorting capabilities have recently become available, enabling the sorting of cells based on their image information. In this review, we specifically highlight practical applications of IFC in four representative areas: cell cycle analysis, protein localization analysis, immunological synapse formation, and the detection of leukemic cells. In addition, particular emphasis is placed on applications that directly contribute to elucidating molecular mechanisms, thereby distinguishing this review from previous general overviews of IFC. IFC enables the estimation of cell cycle phases from large numbers of acquired cellular images using machine learning, thereby allowing more precise cell cycle analysis. Moreover, IFC has been applied to investigate intracellular survival and differentiation signals triggered by external stimuli, to monitor DNA damage responses such as γH2AX foci formation, and more recently, to detect immune synapse formation among interacting cells within large populations and to analyze these interactions at the molecular level. In hematological malignancies, IFC combined with fluorescence in situ hybridization (FISH) enables high-throughput detection of chromosomal abnormalities, such as BCR-ABL1 translocations. These advances demonstrate that IFC provides not only morphological and functional insights but also clinically relevant genomic information at the single-cell level. By summarizing these unique applications, this review aims to complement existing publications and provide researchers with practical insights into how IFC can be implemented in both basic and translational research.

## 1. Introduction

Cells are the fundamental units of life, and obtaining accurate information regarding the state and role of individual cells is essential for understanding biological processes. Traditionally, cell states have been evaluated as average information from a population of multiple cells. However, the increasing need to analyze cell-to-cell variability and transitional states has recently made single-cell level information acquisition paramount [1]. Consequently, various single-cell analysis technologies have been developed, enabling the acquisition of multidimensional data from individual cells through techniques such as single-cell RNA-seq [2], single-cell ATAC-seq [3], CITE-seq [4], and Mass Cytometry [5].

The paradigm that cellular morphology quantitatively encodes its intrinsic physiological and pathological state was first conceptualized in D’Arcy Wentworth Thompson’s landmark 1917 publication, On Growth and Form [6]. This concept has been carried forward and remains fundamental to contemporary life sciences [7,8]. Particularly in blood cells, an expert can identify not only differentiated cells but also cells in intermediate (transitional) states and abnormal cells by appropriately staining a specimen smeared on a glass slide. This indicates that cellular morphological features contain information that allows for classification with a certain degree of accuracy. Imaging Flow Cytometry (IFC) is another technology that enables high-throughput single-cell analysis and has garnered attention as an innovative technique that merges flow cytometry (FCM) with microscopy-based image analysis [9,10].

**Figure 1:**
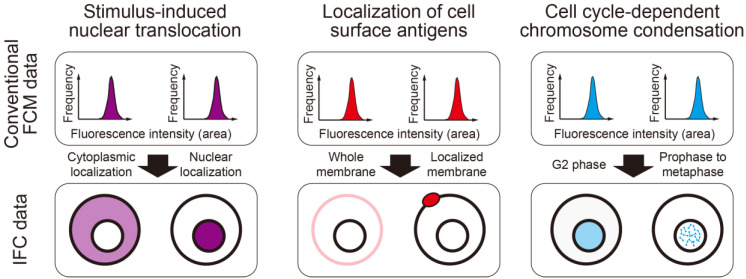
Differences in acquirable data between conventional FCM and IFC. In conventional FCM (**top** panel), cells are discriminated based only on the intensity of fluorescence signals, making it impossible to distinguish between cells with different intracellular localizations of proteins or organelles. In IFC (**bottom** panel), not only fluorescence intensity but also its spatial information is acquired as an image, enabling a more detailed discrimination of cell states.

IFC can acquire not only fluorescence intensity data from fluorescently labeled antibodies or substances that specifically bind to organelles, but also high-precision, large-scale localization data. This allows for the discrimination of cell states based on localization information, which was previously indistinguishable using conventional FCM (Figure 1). Consequently, IFC is utilized in diverse research areas, including the detection of leukemia cells [11,12], cell cycle analysis [13,14,15], detection of chromosomal abnormalities using the FISH method [16,17,18,19,20,21,22,23,24], and evaluation of DNA damage [25,26] based on molecular localization.

Furthermore, with the recent integration of deep learning technologies, new analytical methods that utilize large volumes of single-cell image data have been established. IFC is expected to be particularly useful for single-cell and multi-omics analyses using cell polarity and localization information, which could lead to the identification of unique cell populations that were previously masked [27,28]. Moreover, instruments equipped with both image analysis and cell sorting capabilities are now commercially available, making it possible to sort cells based on morphological features and the localization of proteins and organelles, which was previously impossible to achieve [29,30].

Several reviews on IFC have recently been published [31,32]. While these provide valuable insights into the technical development and broad applications of IFC, the present review specifically emphasizes molecular biology–oriented applications, focusing on how IFC can be applied to dissect cellular mechanisms such as cell cycle regulation, protein localization, immunological synapse formation, and leukemic cell detection.

The present review was prepared by systematically searching PubMed, Web of Science, and Google Scholar databases using the keywords “imaging flow cytometry,” “cell cycle,” “protein localization,” “immunological synapse,” “leukemia,” and “nanoparticle.” Publications up to July 2025 were considered, and additional references were identified through manual screening of cited articles.

## 2. Advances and Current Applications of Imaging Flow Cytometry (IFC)

### 2.1. History of IFC

The concept of IFC was developed from the idea of integrating the high-throughput capability of conventional FCM with the spatial information provided by cellular morphology and subcellular localization [33]. FCM traditionally analyzed cells based on quantitative data, primarily fluorescence intensity and light scatter information [34]. While this was extremely powerful for characterizing cell populations, it could not capture the visual information of individual cells, such as their morphological features or the spatial distribution of molecules. To overcome this limitation, the development of technology to capture images of rapidly moving cells in real-time progressed from the late 1990s to the 2000s [35,36].

In the 2000s, with the introduction of the ImageStream series by Amnis Corporation (now Cytek Biosciences, Fremont, CA, USA), IFC was established as a practical technology capable of acquiring cellular fluorescence images with high speed and precision [37,38]. This opened up new possibilities in life science research by enabling the analysis of cellular morphological features and dynamics that were difficult to detect with conventional FCM. Early IFC systems were developed by combining the fluidic control technology of conventional FCM with high-sensitivity cameras and image acquisition systems. A method was established wherein a high-speed camera continuously captures images of cells passing through a flow cell, which are subsequently digitized and analyzed.

Furthermore, various novel IFC systems are under development at the research-laboratory level, reflecting the significant interest in this field [39,40,41,42,43,44,45,46,47,48].

### 2.2. Current Applications of IFC

Currently, commercially available IFC systems are typified by the ImageStream^®^X Mark II and FlowSight^®^ (both from Cytek Biosciences, formerly Amnis). The ImageStream^®^X Mark II can acquire up to 12 channels of fluorescence images and can analyze tens of thousands of cell images per sample at a high rate. FlowSight^®^ has a more compact and user-friendly design, making it more accessible to standard FCM users. The ImageStream^®^X Mark II combines multiple cameras and an advanced optical system to simultaneously acquire high-resolution cell images, enabling multicolor fluorescence analysis. Its analytical power is applicable to evaluating a wide range of cell biological features, including nuclear shape, granule distribution within the cell, and the co-localization of specific proteins. In particular, the ImageStream^®^X Mark II, with up to 12 fluorescence channels and high-sensitivity detectors, allows for the analysis of more complex cell populations and for the detection of weak fluorescence signals [37].

The ImageStream^®^ was one of the first commercially available IFC instruments. Its graphical user interface is different from that of traditional FCM and microscopes, presenting a slight learning curve. However, it is capable of high-resolution (20×, 40×, and 60×) image acquisition and is equipped with an excellent autofocusing function, brightfield, darkfield, and numerous fluorescence channels. As a result, despite being on the market for nearly 20 years, it still maintains performance that is comparable to or superior to other IFC instruments. Its dedicated analysis software IDEAS^®^ can automatically analyze cellular feature parameters to identify specific populations. Images acquired with the ImageStream^®^X Mark II can be linked to CellProfiler [49,50] for custom analyses, including machine learning. Recently, a machine learning option has been made available within IDEAS^®^, allowing life science researchers without complex coding knowledge to utilize machine learning-based analysis through an intuitive interface.

Another instrument attracting recent attention is the BD FACSDiscover^TM^ S8 Cell Sorter from BD Biosciences (San Jose, CA, USA). This system is groundbreaking in its integration of IFC’s image acquisition capabilities with high-precision cell sorting. This allows for the high-purity sorting of cells based on specific morphological features or molecular localization patterns derived from image information. For example, it is now possible to perform selections that were impossible with previous cell sorter, such as sorting only cells where a fluorescent probe has accumulated in a specific organelle or isolating only cells with abnormal nuclear morphology. The addition of this sorting function is expected to dramatically expand the applications of IFC, enabling more precise cell selection in fields such as disease research, drug discovery, cell therapy, and molecular biology [28].

The BD FACSDiscover^TM^ S8 features an automated startup sequence, making setup easy even for beginners. It can utilize a very large number of fluorescence parameters, and its use of spectral analysis and unmixing makes it easier to resolve issues of fluorescence overlap compared to previous models. Regarding imaging, it has the resolution to distinguish 0.2 µm polystyrene beads from noise and can acquire FSC and SSC images along with information from three fluorescence channels excitable by a 488 nm laser (534 ± 46 nm, 600 ± 60 nm, and 788 ± 225 nm). However, fluorescence compensation cannot be applied to the image-enabled detection channels, so color combinations must be chosen carefully. On the other hand, the user must manually set the image feature parameters of interest. Therefore, to use the sorting function for analyzing a population with specific features, it is advisable to conduct preliminary experiments to sufficiently narrow down the target [29].

Furthermore, VisionSort^TM^ by ThinkCyte (Tokyo, Japan) has emerged, based on a technique called “Ghost Cytometry” [30]. This method uses machine learning to discriminate and sort cells based on their morphological information without generating human-recognizable images. Ghost cytometry combines machine learning technology with optical filtering to rapidly classify and sort cells based on optical features, without performing image acquisition like conventional IFC. This significantly increases the measurement speed compared to traditional IFC and is particularly suitable for experiments involving a large number of cells.

Thermo Fisher Scientific (Waltham, MA, USA) has also launched the Attune^TM^ CytPix Flow Cytometer, which integrates brightfield imaging capabilities into its high-performance and easy-to-set-up compact FCM, the Attune^TM^ NxT Flow Cytometer. The Attune^TM^ CytPix Flow Cytometer facilitates the removal of doublets, which are often difficult to exclude based solely on FCM plot information.

Another recent development is deformation cytometry, which integrates microfluidics with high-speed bright-field imaging to measure cell deformability in real time [51]. This approach enables label-free characterization of size, shape, and mechanical properties such as apparent Young’s modulus at high throughput. A commercial implementation (AcCellerator, Zellmechanik Dresden) incorporates sorting capabilities for label-free cell separation based on mechanical phenotypes, and can also be equipped with fluorescence detection modules to allow simultaneous molecular and mechanical readouts.

In addition, various innovative IFC research projects are being conducted at the laboratory level, indicating the high level of interest in this field.

## 3. Practical Applications of IFC in Basic Medical and Life Science Research

Cells analyzable by IFC are primarily suspension cells, such as blood cells, but adherent cells can also be analyzed after being detached from culture plate or flask and appropriately processed. Here, we describe practical examples of analysis using IFC.

### 3.1. Cell Cycle Analysis

It is a common practice to analyze the cell cycle using FCM by fluorescently labeling the nucleus with a nuclear staining reagent. While FCM can classify cells into G1, S, and G2/M phases based on the signal intensity from the nucleus-bound fluorescent dye, IFC can further refine this classification by adding image information [13,14,15]. Cells undergo dynamic structural changes during mitosis, such as chromosome condensation and segregation. IFC allows for the detailed classification of these events into prophase, metaphase, anaphase, and telophase by visualizing markers like phospho-histone H3 (a G2/M transition marker) or by using antibodies specific to mitotic proteins, such as MPM-2 (Mitotic Protein Monoclonal-2).

However, manually classifying a large number of cells for these M phase subdivisions or subtle morphological changes between G1, S, and G2 phases is extremely labor-intensive and raises issues of objectivity. To solve this problem, analysis combining large-scale cell images acquired by IFC with machine learning [14,15], particularly deep learning [15], has emerged as a promising approach in recent years. In this method, a subset of cells is accurately labeled with their respective cell cycle stages using fluorescence staining, providing ground truth for supervised learning. A model trained on these labeled data can learn to recognize complex morphological features from unstained brightfield and darkfield images. Once trained, the model enables objective and high-throughput classification of tens of thousands of unlabeled cell images with high accuracy [15]. The dedicated analysis software for the ImageStream^®^X Mark II, IDEAS^®^, also offers a machine learning function as an option, allowing researchers without programming knowledge to use this powerful analytical method through an intuitive interface. This enables a more detailed and large-scale analysis of phenomena such as cell cycle arrest at specific stages due to drug responses.

### 3.2. Analysis of Protein Localization

The specific location of proteins within the cell is critically important for cellular function. Conventional FCM was unable to capture phenomena such as the localization or aggregation of proteins within the cell, as it can only measure the total fluorescence intensity of the entire cell. IFC solves this problem and serves as a powerful tool for the detailed analysis of the spatial dynamics of proteins [25,26,28,52].

One example is the analysis of the nuclear translocation of the transcription factor NF-κB, which controls inflammatory responses [53,54]. In its inactive state, NF-κB is sequestered in the cytoplasm through its association with an inhibitory protein, IκB. When cells are stimulated with inflammatory cytokines such as TNF-α, signals from the receptor activate the IKK (IκB kinase) complex. Activated IKK then phosphorylates IκB, which triggers its degradation. Once dissociated from IκB, NF-κB (e.g., the p65 subunit) translocates into the nucleus to induce the transcription of its target genes. Using IFC, this nuclear translocation can be clearly visualized by co-staining cells with a nuclear stain and a fluorochrome-conjugated anti-NF-κB antibody. Furthermore, analysis software can quantify the degree of overlap between the nuclear region and the NF-κB signal as a “Similarity Score,” enabling the precise calculation of the percentage of activated cells (i.e., cells in which NF-κB has translocated into the nucleus) in response to a stimulus.

Other studies have utilized IFC to investigate FOXO1 translocation in human lymphocytes. FOXO1 is a key transcription factor that regulates the development, differentiation, and activation of T and B cells, exerting its function through its localization within the nucleus. In the nucleus, FOXO1 is essential for T and B cell development and for maintaining the stability of regulatory T cells (Tregs) by activating genes such as Foxp3 and Klf2. Conversely, signaling from the T cell receptor (TCR) activates the Akt pathway, which phosphorylates FOXO1, leading to its translocation from the nucleus to the cytoplasm. This translocation suppresses the transcriptional activity of FOXO1, thereby promoting cell proliferation and differentiation, primarily towards an effector T cell (Teff) phenotype. For instance, one study successfully tracked the nucleus-to-cytoplasm translocation of FOXO1 at a single-cell resolution in a human T cell line and peripheral blood mononuclear cells following stimulation with phorbol 12-myristate 13-acetate/ionomycin (PMA/I) [55]. This PMA/I-induced translocation was inhibited by a specific inhibitor of Akt, an upstream kinase of FOXO1. Thus, while conventional Western blotting is limited to bulk-level analysis, IFC enables the investigation of stimulus responsiveness within distinct cell subpopulations.

The analysis of the DNA damage response is another important application of IFC. When DNA double-strand breaks are induced by radiation or drugs, the phosphorylated histone γH2AX accumulates at the damage sites, forming punctate structures called “foci” [25,26]. As a sensitive marker of DNA damage, phosphorylated H2AX (γH2AX) is expected to be applied in the evaluation of genotoxicity and carcinogenicity caused by chemical substances, reactive oxygen species, UV light, and radiation. Furthermore, the detection of γH2AX has recently become known as an indicator for assessing cellular senescence. While conventional FCM could only measure the total cellular fluorescence intensity of γH2AX, IFC allows for the direct counting of the number and brightness of foci formed within the nucleus for each individual cell from its image [25,26]. This enables a more precise evaluation of the degree of DNA damage caused by anticancer drugs and the subsequent repair process.

Thus, IFC provides spatial information on “where and how much” a protein is concentrated as quantitative data, enabling the elucidation of biological phenomena that were impossible to study with conventional methods.

### 3.3. Analysis of the Immunological Synapse

**Figure 2:**
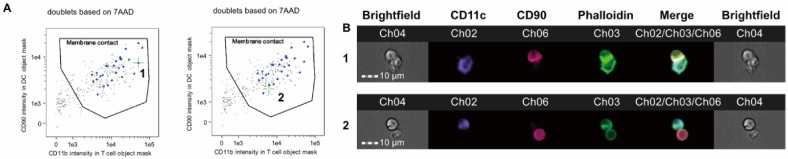
Identification of immune synapse formation by imaging flow cytometry (modified from Juvet et al., J. Vis. Exp., 2017 [60], licensed under CC BY 3.0). Unlike conventional FCM, which only provides population-averaged data, imaging flow cytometry (IFC) enables clear discrimination between simple cell–cell contact and genuine immune synapse formation. Representative doublets of CD4^+^ T cells and dendritic cells are shown. (**A**) By plotting T-cell marker fluorescence (CD90) in the APC object mask against APC marker fluorescence (CD11c) in the T-cell object mask, doublets in actual contact can be gated. Event 1 (left, green crosshairs) represents an immune synapse, while event 2 (right, green crosshairs) represents mere membrane contact without synapse formation. (**B**) Brightfield and fluorescence images of each event. In event 1, actin cytoskeletal rearrangement visualized by phalloidin-FITC accumulates at the T–APC interface, indicating a mature immune synapse. In contrast, event 2 shows membrane contact but lacks actin reorganization, and thus no synapse is formed. Significance: This figure highlights the unique strength of IFC in visualizing and quantifying immune synapses at the single-cell level, providing a functional readout of T-cell activation that bridges molecular signaling studies with clinical applications in transplantation and immunotherapy.

The immunological synapse is a structure of assembled molecules formed at the interface between an immune cell, such as a T cell, and a target cell, like an antigen-presenting cell or a cancer cell, during recognition [56,57]. Within this synapse, molecules like the T cell receptor (TCR) and adhesion molecules dynamically rearrange and accumulate, playing a central role in the initiation and regulation of the immune response [58,59]. In the initial signaling events, TCR stimulation leads to the activation of the Src family kinase Lck via the co-receptors CD4 or CD8. Lck phosphorylates the immunoreceptor tyrosine-based activation motifs (ITAMs) on the CD3 chains within the TCR complex, which serves as the starting point for signal transduction [57]. Subsequently, the kinase ZAP-70 is recruited to the phosphorylated ITAMs and becomes activated. Activated ZAP-70 then phosphorylates the adapter molecules LAT and SLP-76. These serve as a scaffold for the assembly of downstream molecules such as PLCγ1, Vav1, and Grb2, which in turn activate multiple signaling pathways. These molecules form microclusters that accumulate in the center of the central supramolecular activation cluster (cSMAC), thereby amplifying the signal [59]. In addition, sustained T cell activation requires calcium influx from the extracellular space. This calcium influx is maintained by the voltage-gated potassium channel Kv1.3. Kv1.3 channels are redistributed to the IS, where they release potassium ions to maintain a negative membrane potential, thus sustaining calcium influx [58]. Analyzing this complex cell-cell interaction and the spatial arrangement of molecules was impossible with conventional FCM, which only provides average information for a cell population; however, IFC enables clear identification of these events [60], as shown in Figure 2.

In contrast, IFC, with its ability to utilize localization information, is exceptionally well-suited for analyzing the immunological synapse formed through cell-cell contact [61,62]. First, by labeling T cells and target cells with different fluorescent dyes, cell pairs (doublets) where the two have conjugated can be rapidly identified using the principles of FCM. Next, using the image information of the identified cell pairs, the localization of specific proteins at the synaptic interface can be analyzed in detail. This allows for the quantification of the extent to which molecules like the TCR and the adhesion molecule LFA-1 are accumulated (polarized) at the synapse [61]. This enables the statistical evaluation of the T cell activation state and the effects of drugs that modulate its function at the immunological synapse level across a large number of cells. In this way, IFC serves as a powerful method for elucidating the cell-cell communication that forms the basis of the immune response, through a high-throughput combination of images and quantitative data.

### 3.4. Detection of Leukemic Cells

Hematological malignancies such as leukemia are frequently characterized by specific genetic mutations and chromosomal abnormalities, including translocations such as the Philadelphia chromosome. Traditionally, the detection of these abnormalities has relied on fluorescence in situ hybridization (FISH), in which fluorescently labeled DNA probes hybridize to target sequences in fixed cells on glass slides. Although FISH is a well-established and reliable technique, it requires manual microscopic observation and is typically limited to the analysis of a few hundred cells. This limitation makes it difficult to detect rare abnormal cells, such as those associated with minimal residual disease (MRD), which may be present at very low frequencies.

To address this challenge, IFC has been combined with FISH to enable high-throughput and quantitative analysis of chromosomal abnormalities in cell suspensions [16,17,18,19,20,21,22,23,24]. This approach has been applied to detect rare leukemic cells using tens of thousands of individual cell images. For example, in the detection of the BCR-ABL1 fusion gene involved in chronic myeloid leukemia (CML), fluorescent DNA probes targeting the BCR and ABL1 loci are labeled with distinct fluorophores [24]. In normal cells, the green and red signals are spatially separated in the nucleus, whereas in cells harboring the t(9;22) translocation, the signals are colocalized, appearing as a fused signal.

A representative instrument that supports this IFC-based FISH approach is the MI-1000 imaging flow cytometer developed by Sysmex Corporation [18,19]. When used with the proprietary MI FISH Master software, the MI-1000 enables automated and sensitive detection of chromosomal abnormalities in hematologic malignancies. For example, translocations such as BCR-ABL1 can be detected with a sensitivity of 0.1% or lower by analyzing more than 20,000 cells. This capability makes the MI-1000 suitable for identifying rare leukemic cells, including minimal residual disease, in clinical research applications with minimal manual intervention. Due to its ability to acquire large numbers of single-cell images rapidly and perform detailed localization analysis, IFC-based FISH provides a powerful platform not only for the diagnosis of leukemia but also for monitoring treatment response and detecting disease relapse.

Furthermore, combining IFC with deep learning is also being explored for the automated discrimination of leukemic cells [11,12,63]. Well-trained, deep learning-based models have shown the potential to achieve higher discrimination accuracy than human experts [12].

## 4. Future Directions

From its inception to the present day, IFC has contributed to medical and life sciences, including molecular biology, by linking cellular image information with quantitative data. The continued evolution of IFC holds the potential to transform it from a mere analytical tool into an integrated platform that drives new discoveries. Its greatest future potential lies in the fusion of image-based cell sorting with single-cell multi-omics analysis. It is becoming possible to physically sort cells based on image features—such as “protein has translocated to the nucleus,” “an immunological synapse has formed,” or “has an abnormal nuclear morphology”—which was previously only possible based on fluorescence intensity. This will allow for next-generation sequencing analyses, such as single-cell RNA-seq, to be performed exclusively on these sorted populations. This opens a path to directly elucidating the gene expression profiles of rare but functionally critical cell populations that were previously masked within the bulk and could not be distinguished by conventional FCM.

Furthermore, as is already beginning to be implemented, the integration of IFC and machine learning, especially deep learning, is expected to deepen. Currently, cell classification using supervised learning trained by experts is mainstream. In the future, unsupervised learning, where artificial intelligence (AI) autonomously discovers unknown morphological features from massive amounts of cell images and defines new cell states associated with diseases or drug responses, is anticipated to lead to new discoveries. Additionally, the development of AI-driven technologies like ghost cytometry, which directly and rapidly discriminates and sorts cells based on optical features without generating images, is expected to dramatically increase analysis throughput.

These technological innovations will accelerate the transition from basic research to clinical applications. For example, in the detection of circulating tumor cells (CTCs) and minimal residual disease (MRD), combining cell counts with AI-based morphological abnormality scoring could enable more accurate disease monitoring. Moreover, by applying multiple drugs to patient-derived cells and analyzing their responses in detail with IFC (e.g., the formation of DNA damage foci), it is expected to contribute to personalized medicine by selecting the optimal therapeutic drug for each individual patient.

Another promising direction is the use of IFC to investigate cell–nanoparticle interactions. In particular, IFC enables visualization and quantification of nanoparticle uptake and intracellular trafficking, which are difficult to assess with conventional flow cytometry. This approach has also been applied to extracellular vesicles and engineered nanocarriers using single-particle analysis by IFC [64,65]. Such applications are expected to advance the development of safer and more effective nanomedicines.

In summary, IFC is evolving from a conventional cell analysis tool into a “platform for discovery” that integrates advanced AI-driven decision-making with physical sorting capabilities. By directly linking cellular morphology and molecular localization with biological function, IFC holds significant potential to drive new breakthroughs in life sciences and medicine.

## Data Availability

Example IFC image data presented in this review are available upon request.

## Abbreviations

The following abbreviations are used in this manuscript:

AI	Artificial intelligence
CML	Chronic myeloid leukemia
cSMAC	Central supramolecular activation cluster
CTC	Circulating tumor cell
FISH	Fluorescence in situ hybridization
FCM	Flow cytometry
IFC	Imaging flow cytometry
IKK	IκB kinase
ITAM	Immunoreceptor tyrosine-based activation motif
MPM-2	Mitotic Protein Monoclonal-2
MRD	Minimal residual disease
PMA/I	Phorbol 12-myristate 13-acetate/ionomycin
TCR	T cell receptor
Teff	Effector T cell
Treg	Regulatory T cell
